# An exceptional P-H phosphonite: Biphenyl-2,2'-bisfenchylchlorophosphite and derived ligands (BIFOPs) in enantioselective copper-catalyzed 1,4-additions

**DOI:** 10.1186/1860-5397-1-6

**Published:** 2005-08-26

**Authors:** T Kop-Weiershausen, J Lex, J-M Neudörfl, B Goldfuss

**Affiliations:** 1 Institut für Organische Chemie, Universität zu Köln, Greinstrasse 4, 50939 Köln, Germany

**Keywords:** phosphorus ligands, chirality, biaryls, asymmetric conjugate additions, phosphoramidites, phosphites, phosphonites, X-ray analyses

## Abstract

Biphenyl-2,2'-bisfenchol (BIFOL) based chlorophosphite, BIFOP-Cl, exhibits surprisingly high stabilities against hydrolysis as well as hydridic and organometallic nucleophiles. Chloride substitution in BIFOP-Cl proceeds only under drastic conditions. New enantiopure, sterically demanding phosphorus ligands such as a phosphoramidite, a phosphite and a P-H phosphonite (BIFOP-H) are hereby accessible. In enantioselective Cu-catalyzed 1,4-additions of ZnEt_2_ to 2-cyclohexen-1-one, this P-H phosphonite (yielding 65% ee) exceeds even the corresponding phosphite and phosphoramidite.

## Introduction

**Scheme 1:**
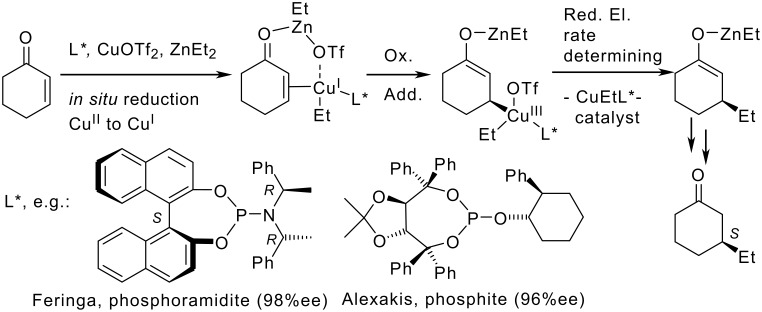
Monodentate phosphorus ligands, e.g. BINOL-based phosphoramidites or TADDOL-based phosphites, are highly efficient in copper catalyzed enantioselective conjugate additions.

Chiral monodentate phosphorus ligands with C_2_-symmetric diol backbones, e.g. with the prominent BINOLs or TADDOLs, are fundamental for the construction of efficient enantioselective transition metal catalysts, especially for copper-catalyzed 1,4-additions. [1–18] Such asymmetric conjugate additions of diethylzinc to enones are often highly enantioselective, especially with phosphoramidites (amidophosphites) and phosphites. [2,19–41] These chiral ligands (L*) exhibit large steric demands and good metal to ligand back bonding abilities. Such ligands generate active R-Cu^I^-L* catalysts and support the rate determining reductive elimination in the catalytic cycle (Scheme 1). [42–47]

**Scheme 2:**
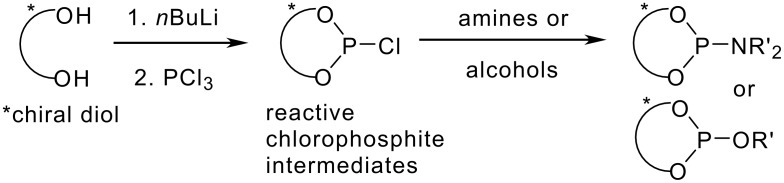
Modular phosphoramidites (R= NR'2) or phosphites (R= OR') from reactive chlorophosphite intermediates.

Common basis for diol-based phosphoramidites and phosphites are highly reactive chlorophosphites. [48–51] These intermediates are converted usually *in situ* with amines or alcohols to the modular, enantiopure ligands (Scheme 2). [2,19–41].

Modular fencholates were recently applied in chiral organolithium reagents [52–58] and in organozinc [59–62] as well as in organopalladium catalysts [63–67] to study origins of enantioselectivities in C-C-couplings. The rigid, terpene-based bicyclo[2.2.1]heptane unit enables efficient, stereoselective access to crystalline diol ligands such as BIFOL (*bi*phenyl-bis*f*ench*ol*), [68–72] which we here apply for constructions of new BIFOL-based, phosphorus ligands, i.e. *bi*phenylbis*f*ench*o*l*p*hosphanes (BIFOPs). BIFOPs with high steric demand and good acceptor abilities are then employed in enantioslective Cu-catalyzed 1,4-additions.

## Results and Discussion

**Figure 1:**
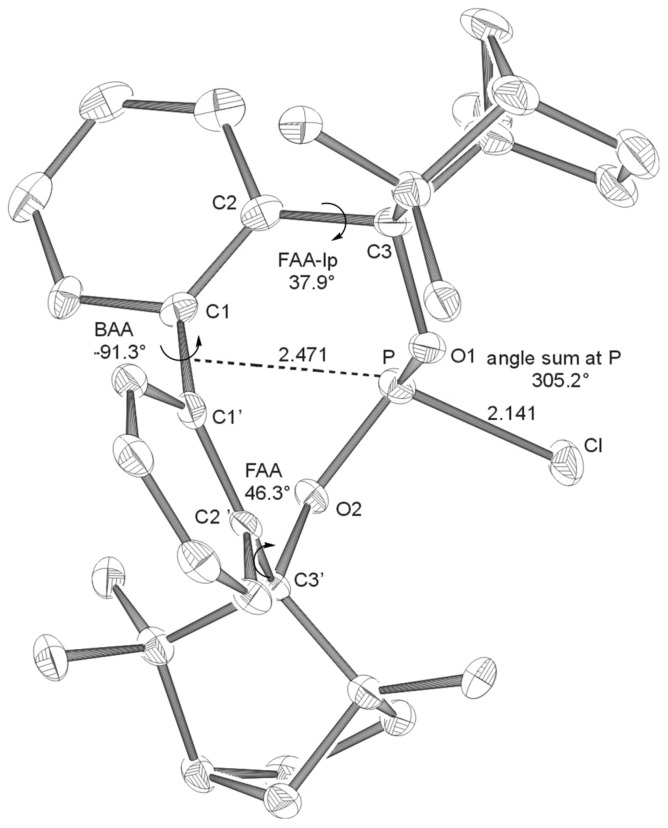
X-ray crystal structure of **BIFOP-Cl** (**1**). Distances are given in Å. (BAA = biaryl angle between C_2_-C_1_-C_1_'-C_2_'; FAA-lp = fenchyl-aryl dihedral angle between C_1_-C_2_-C_3_-O_1_; FAA = fenchyl-aryl dihedral angle between C_1_'-C_2_'-C_3_'-O_2_). The probability of ellipsoids is 40% (CDC 270531).

**Figure 2:**
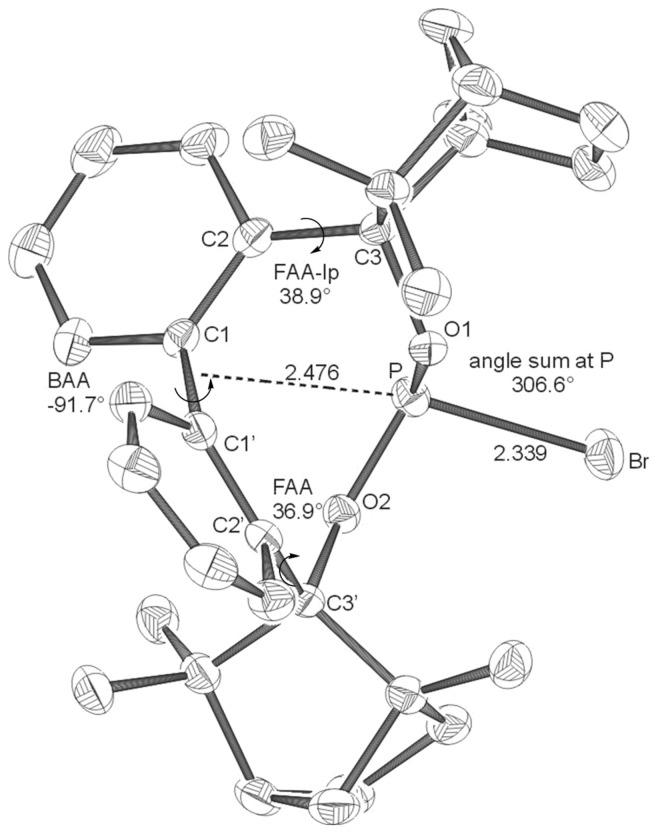
X-ray structures of **BIFOP-Br** (**2**). Distances are given in Å. (BAA = biaryl angle between C_2_-C_1_-C_1_'-C_2_'; FAA-lp = fenchyl-aryl dihedral angle between C_1_-C_2_-C_3_-O_1_; FAA = fenchyl-aryl dihedral angle between C_1_'-C_2_'-C_3_'-O_2_). The probability of ellipsoids is 30% (CCDC 270532).

**Scheme 3:**
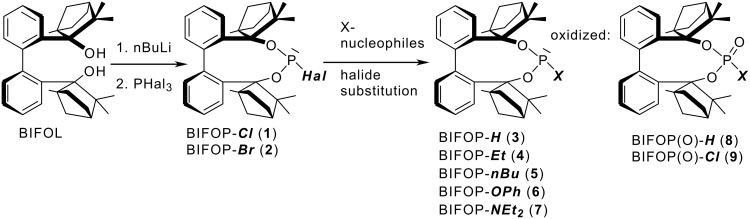
Synthesis of biphenyl-2,2'-bisfenchol (BIFOL) based phosphane derivatives (BIFOPs).

Coupling of bis-lithiated biphenyl-2,2'-bisfenchol (BIFOL), synthesized from 2,2'-dilithiobiphenyl and (-)-fenchone, [68–72] with PCl_3_ or PBr_3_ yields the enantiopure halophosphites BIFOP-Cl, **1** (62% yield) and BIFOP-Br, **2** (69% yield, Scheme 3), which are air stable (no hydrolysis or oxidation) over weeks, crystalline and analyzable via X-ray diffraction. [73–76] In close analogy to the hydrogen bonded *M*-BIFOL, [68–72] only *minus*-(*M*)-conformations of biaryl axes are found in these BIFOP (*bi*phenylbis*f*ench*o*l*p*hosphane) halides (Figure 1 and Figure 2).

**Figure 3:**
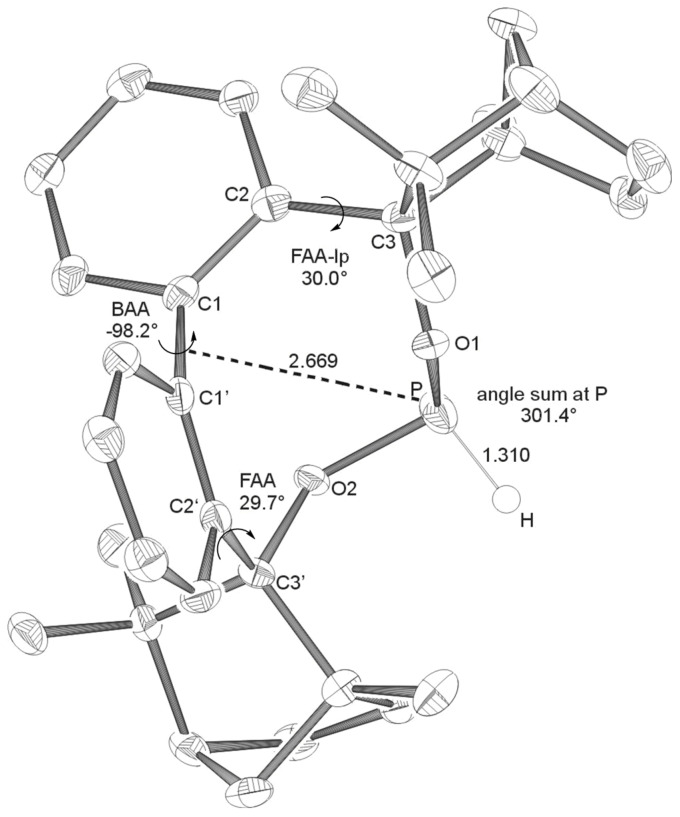
X-ray structures of **BIFOP-H** (**3**). Distances are given in Å. (BAA = biaryl angle between C_2_-C_1_-C_1_'-C_2_'; FAA-lp = fenchyl-aryl dihedral angle between C_1_-C_2_-C_3_-O_1_; FAA = fenchyl-aryl dihedral angle between C_1_'-C_2_'-C_3_'-O_2_). The probability of ellipsoids is 40% (CCDC 270533).

**Figure 4:**
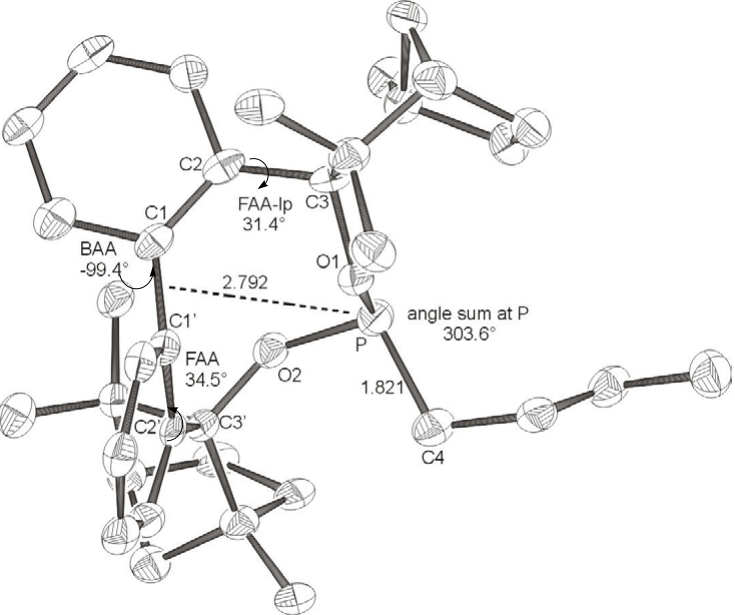
X-ray structures of **BIFOP-*n*Bu** (**5**). Distances are given in Å. (BAA = biaryl angle between C_2_-C_1_-C_1_'-C_2_'; FAA-lp = fenchyl-aryl dihedral angle between C_1_-C_2_-C_3_-O_1_; FAA = fenchyl-aryl dihedral angle between C_1_'-C_2_'-C_3_'-O_2_). The probability of ellipsoids is 40% (CCDC 270534).

**Figure 5:**
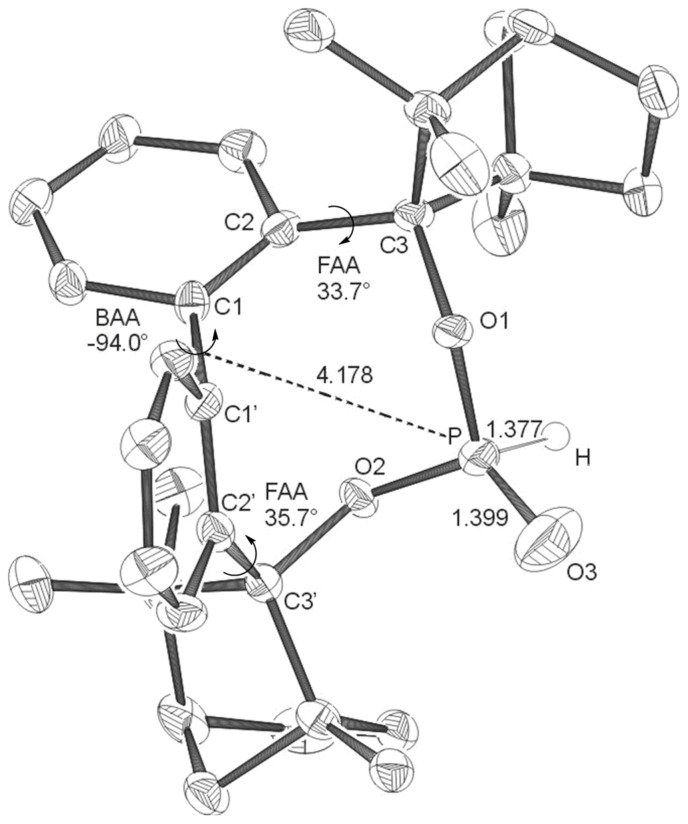
X-ray structures of **BIFOP(O)-H** (**8**). Distances are given in Å. (BAA = biaryl angle between C_2_-C_1_-C_1_'-C_2_'; FAA-lp = fenchyl-aryl dihedral angle between C_1_-C_2_-C_3_-O_1_; FAA = fenchyl-aryl dihedral angle between C_1_'-C_2_'-C_3_'-O_2_). The probability of ellipsoids is 40% (CCDC 270537).

**Figure 6:**
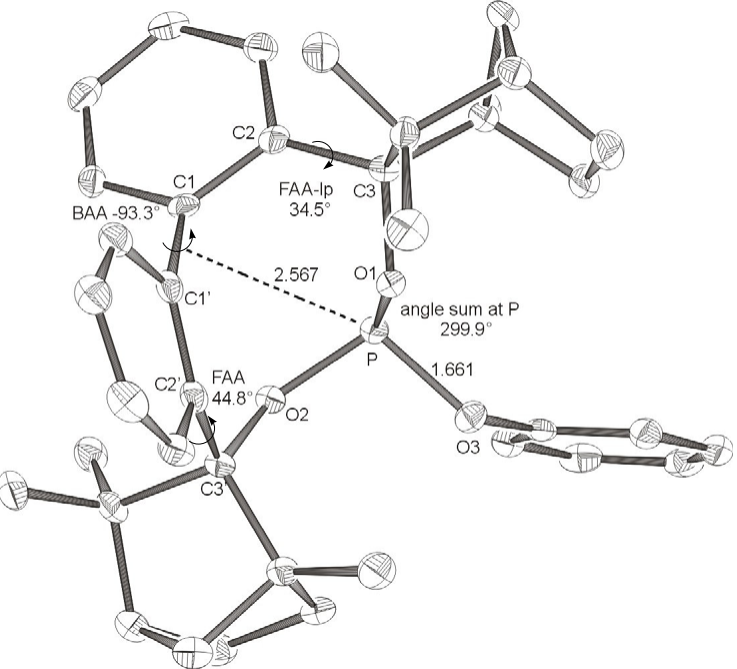
X-ray structures of phosphite **BIFOP-OPh** (**6**). Distances are given in Å. (BAA = biaryl angle between C_2_-C_1_-C_1_'-C_2_'; FAA-lp = fenchyl-aryl dihedral angle between C_1_-C_2_-C_3_-O_1_; FAA = fenchyl-aryl dihedral angle between C_1_'-C_2_'-C_3_'-O_2_). The probability of ellipsoids is 40% (CCDC 270535).

**Figure 7:**
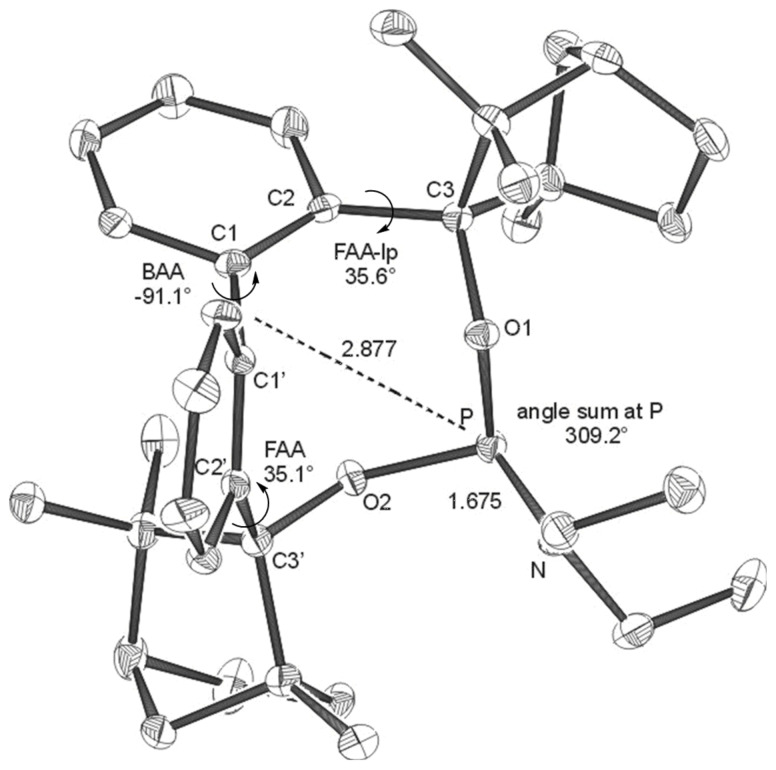
X-ray structures of phosphoramidite **BIFOP-NEt2** (**7**). Distances are given in Å. (BAA = biaryl angle between C_2_-C_1_-C_1_'-C_1_'; FAA-lp = fenchyl-aryl dihedral angle between C_1_-C_2_-C_3_-O_1_; FAA = fenchyl-aryl dihedral angle between C_1_'-C_2_'-C_3_'-O_2_). The probability of ellipsoids is 40% (CCDC 270536).

**Figure 8:**
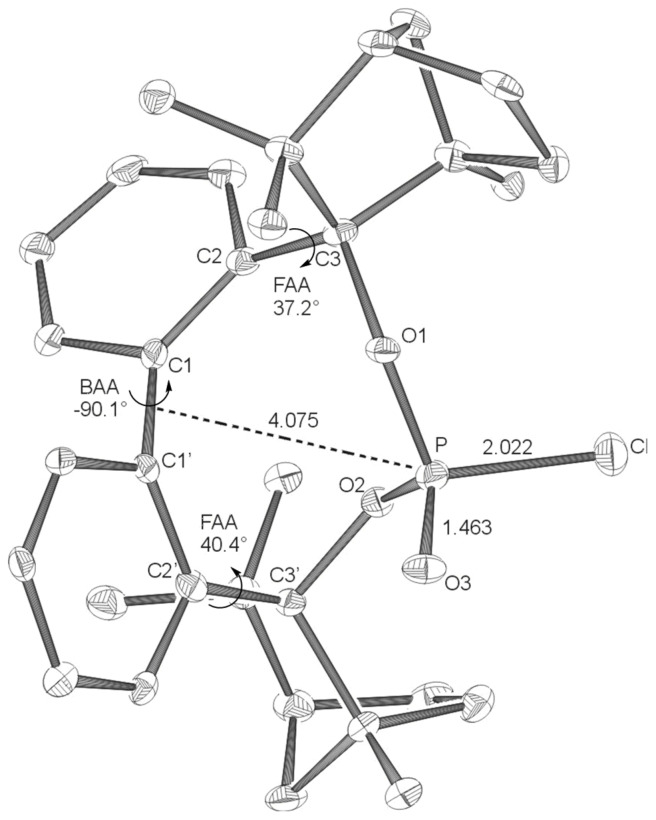
X-ray structures of **BIFOP(O)-Cl** (**9**). Distances are given in Å. (BAA = biaryl angle between C_2_-C_1_-C_1_'-C_2_'; FAA-lp = fenchyl-aryl dihedral angle between C_1_-C_2_-C_3_-O_1_; FAA = fenchyl-aryl dihedral angle between C_1_'-C_2_'-C_3_'-O_2_). The probability of ellipsoids is 40% (CCDC 270538).

Surprisingly, the halophosphites **1** (BIFOP-Cl) and **2** (BIFOP-Br) are very reluctant in reactions with various nucleophilic reagents to give halide substitution (Table 1). [77–78] No nucleophilic substitution [2,19–41] is observed for **1** in treatments with equimolar suspensions of LiAlH_4_ in hexanes or THF at 25°C for 3 h (Table 1, entries 1 and 2). Only elevated temperatures (69°C), longer reaction times (12 h) and an excess of LiAlH_4_ yields the P-H phosphonite BIFOP-H, **3** (79% yield, Table 1, entry 3, Figure 3). Even the strong C-nucleophilic reagents methyllithium, ethyllithium, *n*-butyllithium and *t*-butyllithium gave no expected conversions at low temperatures. Ethylation of BIFOP-Cl (**1**) yielding BIFOP-Et (**4**) was observed with ethyllithium only at elevated temperatures (Table 1, entry 7) or with a large excess of diethylzinc at room temperature (Table 1, entry 13). Similarly, **1** was converted to BIFOP-*n*Bu (**5**, Figure 4) only with an excess of *n*-BuLi at elevated temperatures. The resistance of BIFOP-Cl (**1**) to O-and N-nucleophiles is apparent from reactions with H_2_O, LiOPh and LiNEt_2_. While no hydrolysis of **1** is observed at ambient temperature, only reflux and basic conditions (KOH) yield complete hydrolysis of **1** to BIFOP(O)-H, **8** (98%, Table 1, entry 14, Figure 5). [79–80] The phosphite BIFOP-OPh, **6** (40%, Figure 6) and the phosphoramidite BIFOP-NEt_2_, **7** (47%, Figure 7) are accessible from **1** only at elevated temperatures with LiOPh or LiNEt_2_. The oxo-derivative BIFOP(O)-Cl (**9**) is synthesized by coupling of BIFOL with POCl_3_ (65%, Figure 8).

**Table 1:** Reactivity of BIFOP-Cl (**1**) with various nucleophilic reagents.

entry	Reagent	**1** : reagent	conditions	yield ^a^

1	LiAlH_4_	1 : 1	Rt, 3 h, THF	-^b^
2	LiAlH_4_	1 : 1	Rt, 3 h, hexanes	-^b^
3	LiAlH_4_	1 : 2.5	reflux, 12 h, hexanes	**3**(79%)
4	MeLi	1 : 1.2^c^	-78°C, hexanes, Et_2_O^d^	-^b^
5	MeLi	1 : 5^c^	reflux, 24 h hexanes, Et_2_O	-^b^
6	EtLi	1 : 1.2^c^	-78°C, hexanes, benzene^d^	-^b^
7	EtLi	1 : 5^c^	reflux, 24 h, hexanes, benzene	**4** (63%)
8	nBuLi	1 : 1.2	-78°C-rt, hexanes^d^	-^b^
9	nBuLi	1 : 5	reflux, 24 h, hexanes	**5** (74%)
10	tBuLi	1 : 1.2	-78°C-rt, hexanes^d^	-^b^
11	tBuLi	1 : 5	reflux, 48 h, hexanes	-^b^
12	ZnEt_2_	1 : 2	rt, 2 h, toluene	-^b^
13	ZnEt_2_	1 : 140	rt, 2 h, toluene	**4** (89%)
14	H_2_O	1 : 110	rt, 3 h	-^b^
15	H_2_O/KOH	1 : 220^e^	reflux, 5 days	**8** (98%)
16	LiOPh	1 : 5	-78°C-rt, hexanes^d^	-^b^
17	LiOPh	1 : 5	reflux, 24 h, hexanes	**6** (47%)
18	LiNEt_2_	1 : 5	-78°C-rt, hexanes^d^	-^b^
19	LiNEt_2_	1 : 5	reflux, 24 h, hexanes	**7** (47%)

^a^ The reaction of **1** (δ^31^P: 154.4) was monitored by ^31^P-NMR-spectroscopy; isolated yields are given; ^b^ Only pure BIFOP-Cl (**1**) was recovered (> 91%); ^c^ MeLi 1.6 M solution in diethylether; EtLi 0.5 M solution in benzene/cyclohexane (90/10); ^d^ Reactions were performed at -78°C for 3 h and subsequently at rt for 3 h; ^e^ With KOH (1 M, 0.01 g, 0.18 mmol) in water.

**Scheme 4:**
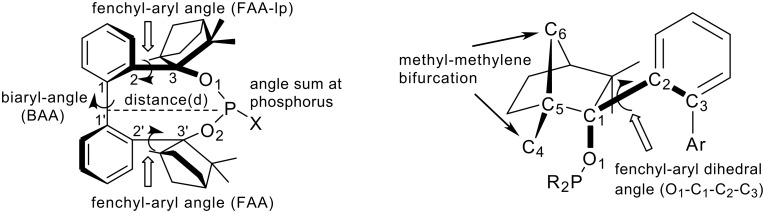
Geometries of BIFOP-systems with respect to biaryl dihedral angles (C_2_-C_1_-C_1’_-C_2’_, BAA), fenchyl-aryl dihedral angles (C_1_-C_2_-C_3_-O_1_) on the lone pair-side of phosphorus (FAA-lp) and at the substituent (X) side (FAA), the pyramidality of phosphorus measured as angle sum and the distance of phosphorus to the biaryl axis (C_1_-C_1’_).

The high steric demand of the embedding fenchane units provides explanations for the unexpectedly low reactivity of the > P-Cl moiety in BIFOP-Cl (**1**). The geometries of all BIFOP-derivatives are remarkable with respect to their biaryl-angles, the fenchyl-aryl-angles, the pyramidality at the phosphorus atoms as well as the positions of the phosphorus atom in the hydrophobic fenchane cavities (Scheme 4, Table 2).

**Table 2:** X-ray structure geometries (cf. Scheme 4) and computed energies of BIFOL and BIFOP-X (**1**–**7**).

BIFOP (-***X***)	E_rel_ (kcal mol^-1^)^a^	BAA (°) ^b^	angle sum (°)^c^	FAA-lp (°)	FAA (°)	d (Å)^d^

*M*-BIFOL	+12.5	-95.0 ^e^	-	(25)	(31)	-
**1** (-*Cl*)	+28.4	-91.3	305.2	37.9	46.3	2.471
**2** (-*Br*)	+35. 0	-91.7	306.6	38.9	36.9	2.476
**3** (-*H*)	+27.9	-98.2	301.4	30.0	29.7	2.669
**5** (-*nBu*)	+26.3	-99.4	303.6	31.4	34.5	2.792
**6** (-*OPh*)	+27.7	-93.3	300.0	34.5	44.8	2.567
**7** (-*NEt*_2_)	+22.5	-91.1	309.2	35.8	35.1	2.877

^a^ Relative Destabilization of *plus* (*P*) conformations according to B3LYP/6-31G*//PM3 computations; ^b^ Biaryl dihedral angle between C2-C1-C1'-C2' atoms (BAA) in degree; ^c^ Angle sum at phosphorus atom (pyramydality) in degree; ^d^ Distance (d) between phosphorus atoms and the center of the biaryl axis (C1-C1'); ^e^ Hydrogend bonded *M*-conformer.

**Figure 9:**
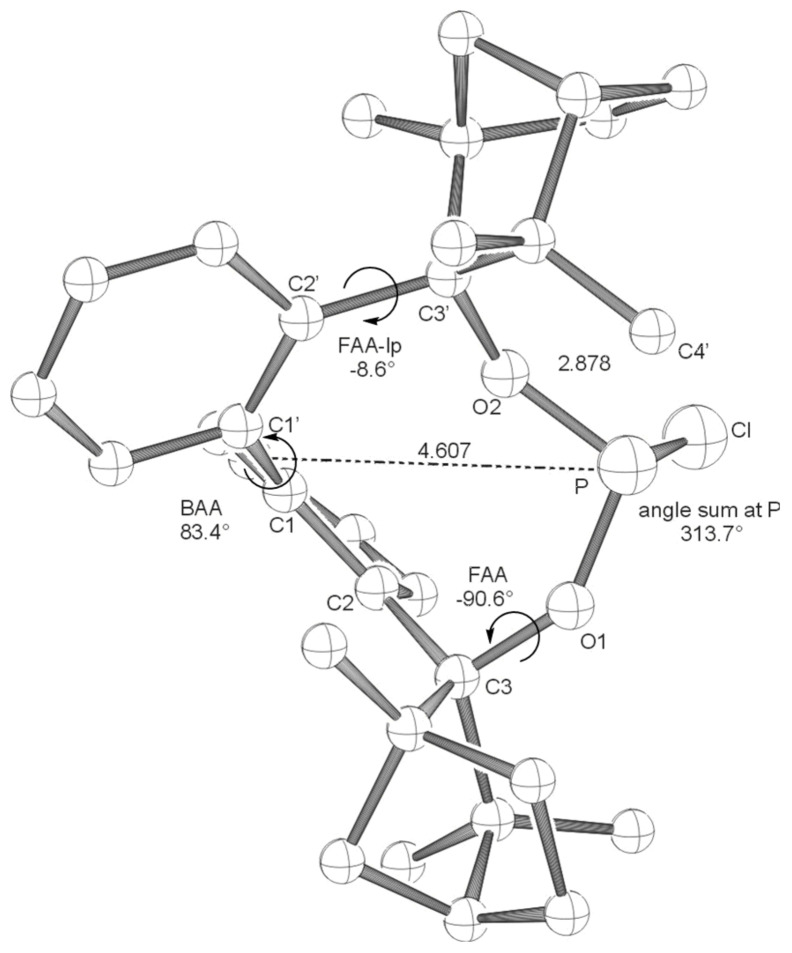
Computed geometry (PM3) of *plus*-(*P*)-**BIFOP-Cl** with *unnatural plus*-(*P*)-biaryl conformation. Distances are given in Å. (BAA = biaryl angle between C_2_-C_1_-C_1_'-C_2_'; FAA-lp = fenchyl-aryl dihedral angle between C_1_-C_2_-C_3_-O_1_; FAA = fenchyl-aryl dihedral angle between C_1_'-C_2_'-C_3_'-O_2_).

A strong preference for the *minus* (*M*)-biaryl conformation was found in BIFOL (Scheme 3) and was attributed to hydrogen bond linked chiral fenchole units, in the solide state and in solution. [68–72] Similarly, all phosphorus linked BIFOPs exhibit *M*-biarly axes with dihedral angles varying from -91° to -99° (Scheme 4, Table 2). The alternative *plus* (*P*)-conformations were not found experimentally, they are computed to be disfavored by ca. 20 to 35 kcal mol^-1^ (Table 2). As in *M*-BIFOL, [68–72] the strong destabilization of these *plus*-(*P*)-conformations arise from steric repulsion of *endo*-oriented fenchane units in BIFOPs, *endo*-methyl groups are close to the phosphorus atoms (Figure 9).

Free rotation around the fenchyl aryl bonds is hindered in aryl fenchol derivaties by the methyl-methylene-bifurcation of the fenchane scaffolds (Scheme 4). These fenchyl aryl dihedral angles (O_1_-C_2_-C_3_-C_1_, FAA, Scheme 4) are crucial for the "bite" of the chiral diol unit and are constrained between 30° and 46° (Table 2), similar to fenchyl aryl angles previously analyzed in lithium fencholates. [54–55] In BIFOL, the asymmetry of the H-bond gives rise to two different fenchyl aryl angles (25° and 31°, Table 2). [68–72] Likewise, the pyramidality at the phosphorus atoms in BIFOPs distorts the inherent C_2_-symmetry of the biphenylbisfenchol units to asymmetry (C_1_). Fenchanes close to the phosphorus lone pairs (with FAA-lp) can be differentiated from fenchanes close to substituents at phosphorus (with FAA, Scheme 4). The difference between these two FAA dihedral angles is a meassure for the BIFOP-asymmetry, which is small for BIFOP-H (**3**) and BIFOP-NEt_2_ (**7**), but large for BIFOP-Cl (**1**) and BIFOP-OPh (**6**, Table 2).

The phosphorus atoms, essential for coordination to (late transition) metals, exhibit slightly different degrees of pyramidality, as it is measured by angle sums (planarity) from 300° to 309° (Table 2, Scheme 4). The degree of encapsulation of the phosphorus atoms by the fenchane units is measured by the distance (d) of the phosphorus atoms to the center of the biaryl axes (C_1_-C_1'_, Scheme 4). The tightest encapsulation and fenchane embedding of phosphorus atoms is apparent for the halophosphites BIFOP-Cl (**1**, 2.471 Å) and BIFOP-Br (**2**, 2.476 Å, Table 2), explaining their unusual low reactivity (Table 1).

**Scheme 5:**
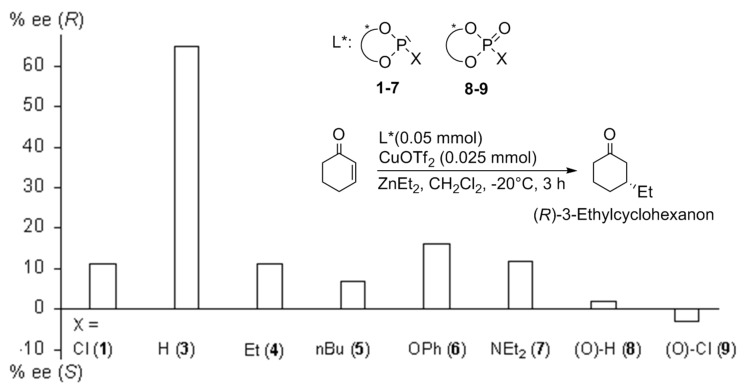
Biphenyl-2,2'-bisfenchol based phosphanes (BIFOPs) as chiral ligands in enantioselective Cu-catalyzed 1,4-additions.

Cu-catalyzed, enantioselecitve 1,4-additions of diethylzinc to 2-cyclohexene-1-one were employed as test reactions for the monodentate phosphorus ligands BIFOPs **1** and **3–7** as well as the oxo-derivatives **8** and **9** (Table 3, Scheme 5).

**Table 3:** Enantioselective Cu-catalyzed 1,4-additions of diethylzinc to 2-cyclohexen-1-one (cf. Scheme 5).^a^

L*, i.e. BIFOP(O)-***X***	substituent ***X***	yield (%)^b^	%ee (config.)^c^

**1** ^d^	Cl	98 ^d^	11 (*R*) ^d^
**3**	H	92	65 (*R*)
**4**	Et	98	11 (*R*)
**5**	*n*Bu	97	7 (*R*)
**6**	OPh	73	16 (*R*)
**7**	NEt_2_	98	12 (*R*)
**8**	(O)-H	89	2 (*R*)
**9**	(O)-Cl	87	3 (*S*)

^a^ Reaction conditions: -20°C, 3 h in CH_2_Cl_2_; L*:Cu(OTf)_2_ ratio (2:1); ^b^ Yield determined by GC; ^c^ The ee's are determined by GC with the chiral column lipodex E 0.2 μm, 50 m, 0.25 mm; ^d^ Chlorophosphite **1** was converted to P-Et phosphonite BIFOP-Et, **4**.

**Scheme 6:**
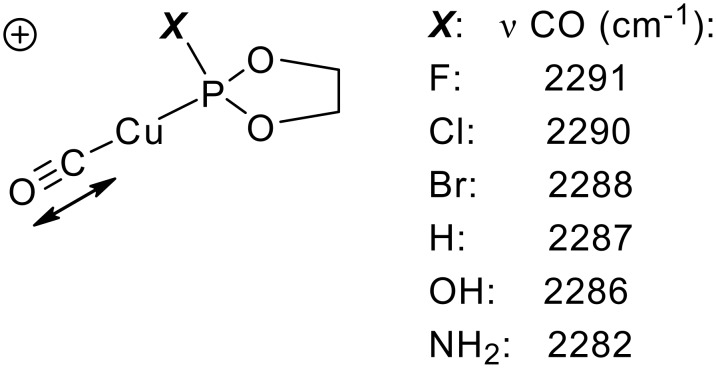
Anharmonic B3LYP/6-31G^*^(C,H,N,O,F,Cl,Br) /SDD(Cu) CO-stretching frequencies to assess metal to ligand back bonding characteristics.

The low reactivity of the chlorophosphite **1** with metal organic nucleophiles (Table 1) points to its potential suitability as ligand for late, electron-rich transition metals, such as Cu^I^. The rate determining reductive elimination was expected to be favored by the good metal to ligand back bonding properties of the σ*(P-Cl) acceptor as is well established in phosphites, i.e. σ*(P-OR), and phosphoramidites, i.e. σ*(P-NR_2_). Computed anharmonic CO-frequencies on Cu^I^-model complexes indeed point to highest νCO stretching frequencies for P-ligands with strongest acceptor character, i.e. the halophosphites (Scheme 6). [81–85]

However, under catalysis conditions, BIFOP-Cl (**1**) converts to the ethylphosphonite BIFOP-Et (**4**), which yields *R*-3-ethylcyclohexanone with 11% ee (Table 3). Apparently, the higher Lewis acidity of organozincs supports faster nucleophilic substitution in (**1**) than with organolithiums (Table 1). Unprecedented however is the P-H phosphonite BIFOP-H (**3**), which yields with 65 % ee a much higher enantioselectivity than the corresponding phosphite (BIFOL-OPh, 16%ee) and phosphoramidite (BIFOL-NEt_2_, 12%ee, Table 3). A good back bonding characteristic between halophosphites and phosphites is indeed apparent for the P-H unit (Scheme 6). [86–87] The relative high enantioselectivity of **3** is remarkable, as the asymmetry of **3**, measured from the difference of its fenchyl-aryl angles, is rather small (FAA-lp = 30.0° vs. FAA = 29.7°, Table 2). The phosphorus atom in **3** is only slightly encapsulated by the fenchane moieties, due to the rather long *d*-distance (Scheme 4, Table 2, 2.7 Å) and the low steric shielding by the H-atom. Indeed, P-H phopshonite **3** coordinates tightly to Cu^II^-ions. Metal free BIFOP-H (**3**) gives in CDCl_3_ a ^31^P-NMR signal at δ = 139.6 with a ^1^J (P,H) coupling of 214.5 Hz. With half of an equivalent of Cu(OTf)_2_, no free **1** is detectable, only a [(**1**)_2_Cu(OTf)_2_] complex is evident from a ^31^P-NMR signal at δ = 81.1 with a stronger ^1^J (P,H) coupling of 299.5 Hz. [86,89]

## Conclusion

The large steric demand of embedding fenchane units makes phosphorus atoms in BIFOPs hardly accessible by nucleophilic reagents and leads to an unusually high stability, e.g. for the chlorophosphite BIFOP-Cl (**1**). While **1** converts to the P-Et phosphonite BIFOP-Et (**4**) during Cu-catalyzed 1,4-additions of diethylzinc to cyclohexenone, the P-H phosphonite BIFOP-H (**3**) is stable and gives even a higher enantioselectivity than a corresponding phosphite or phosphoramidite. Hence, the large steric demand and the relatively low accessibility of the phosphorus atoms in biphenyl-2,2'-bisfenchylphosphites (BIFOPs) founds the special suitability for BIFOP-H (**3**) as P-H phosphonite ligand in transition metal catalysis. This points to promising applications of **3** or analogue P-H ligands in enantioselective catalysis.

## Supporting Information

File 1 Experimental details
