# Supplementary material for: An exceptional P-H phosphonite: Biphenyl-2,2'-bisfenchylchlorophosphite and derived ligands (BIFOPs) in enantioselective copper-catalyzed 1,4-additions
Source: Beilstein J Org Chem. 2005 Aug 26;1:6. doi: 10.1186/1860-5397-1-6 (PMC1399454; doi:10.1186/1860-5397-1-6)
Supplement: File 1 — Experimental details [file Beilstein_J_Org_Chem-01-06-s001.doc]

**Experimental Section**

**General Remarks:** ^1^H-, ^13^C- and ^31^P-NMR spectra were recorded with a Bruker Avance DPX 300 (300 Hz). Chemical shifts are given relative to TMS, CDCl_3_; Melting points (mp) were determined using a Stuart Scientific melting point apparatus SMP3 and are uncorrected; Analytical thin-layer chromatography (TCL) was carried out on Merck pre-coated 0.25 mm thick plates of silica gel 60 F_254_; Gravity chromatography was carried out using Merck Kieselgel 60; Enantiomeric excesses for the conjugate addition product 3-ethylcyclohexanone were measured on a Hewlett Packard HP 6890 GC (chiral capillary column: Lipodex E, 0.2 µm, 50 m, 0.25 mm); Optical rotations were measured with a IBZ, Messtechnik POLAR LµP-WR polarimeter, using a cell of 1 dm path length. Dry THF was distilled from sodium in a recycling still using benzophenone as indicator. Other solvents were purified by standard techniques.^[15]^ The reactions were carried out under dry argon; X-ray analysis were recorded with Nonius Kappa CCD diffractometer (Mo-K_α_; wavelength λ = 0.71073). CCDC **270531** - CCDC **270538** contain the supplementary crystallographic data for this paper. These data can be obtained free of charge via [*www.ccdc.cam.ac.uk/conts/retrieving.html*](http://www.ccdc.cam.ac.uk/conts/retrieving.html) (or from the CCDC, 12 Union Road, Cambridge CB2 1EZ, UK; fax: +44 1223 336033; e-mail: deposit@ccdc.cam.ac.uk).

**Synthesis of biphenyl-2,2'-bisfencholphosphanes (BIFOPs)**

**BIFOP-Cl (1)**: To a stirred solution of 1.05 g biphenyl-2,2'-bisfenchol (BIFOL,^[8]^ 2.3 mmol) in abs. THF (10 ml) 2.81 ml of n-butyllithium (1.6 M, , 4.6 mmol) were slowly added at -20°C. The mixture was stirred for 30 min. at -20°C, then for 1 h at room temperature. After re-cooling to -20°C, 0.3 ml of freshly distilled PCl_3_ (2.3 mmol) were added slowly and the reaction mixture was stirred for 48 h at room temperature. The solvent was removed in vacuo. The resulting cloudy residue was stirred with 10 ml of toluene for 1 h, then LiCl was filtered off through celite. Purification by flash chromatography (diethylether/hexane, 1/4) and recrystallization from diethylether/CH_2_Cl_2_ furnished the title compound **1** as colorless crystals 0.745 g (62%). Analytic and spectroscopic data of **1**: mp: 147°C; [α] -17.25 (2.8 M in hexane); ^31^P-NMR (CDCl_3_): δ 154.4; ^1^H-NMR (CDCl_3_): δ 0.07 (3H,s), 0.30 (3H,s), 0.65 (3H,s), 0.83 (3H,s), 1.10–1.61 (10H,m), 1.72 (3H,m), 2.24 (1H,d), 2.41 (1H,d), 2.48 – 2.67 (2H,m), 6.68 (1H, d), 6.97 (1H,t), 7.14‑7.22 (2H,m), 7.50 (1H,d), 7.62 (1H,d). ^13^C-NMR (CDCl_3_): δ 15.2, 16.1, 22.2, 22.7, 23.5, 23.9, 24.1, 25.4, 26.4, 26.7, 32.6, 38.3, 42.4, 42.6, 48.8, 49.0, 58.3, 58.7, 123.9, 125.8, 131.8, 133.9, 134.4, 143.8.

**X-ray crystal data of 1**: C_32_H_40_ClO_2_P*CH_2_Cl_2_; M_r_ 607.99; space group: P 2_1_2_1_2_1_ orthorohombic;
a = 10.6688(2) Å, b = 15.3190(3) Å, c = 18.4312(5) Å; V = 3012.31(12) Å^3^; Z = 4;
T = 100(2) K; µ = 0.387 mm^-1^; reflections total: 10475, unique: 4911, observed: 4551 (*I*>2σ(*I*)); R1 = 0.0299, wR2 = 0.0670; GOF = 1.027.


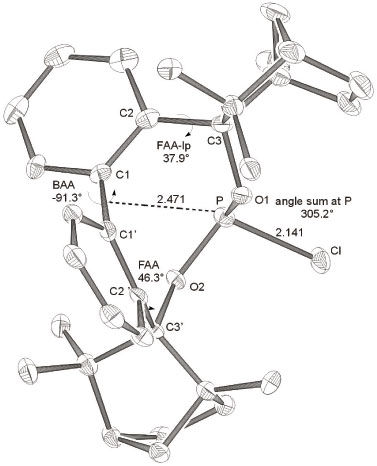


**Figure 1**. X-ray crystal structure of **BIFOP-Cl** (**1**). Distances are given in Å. (BAA = biaryl angle between C2-C1-C1’-C2’; FAA-lp = fenchyl-aryl dihedral angle between C1-C2-C3-O1; FAA = fenchyl-aryl dihedral angle between C1’-C2’-C3’-O2). The probability of ellipsoids is 40% (CDC 270531).

**BIFOP-Br (2)**: To a stirred solution of 0.917 g biphenyl-2,2'-bisfenchol (BIFOL,^[8]^ 2 mmol) in 10 ml abs. THF 2.45 ml of *n*-butyllithium (1.6 M, 4 mmol) were added slowly at -20°C. The mixture was stirred for 30 min. at -20°C, then for 1 h at room temperature. After cooling to -20 °C, 0.19 ml of PBr_3_ (2 mmol) were added slowly and the reaction mixture was stirred for 48 h at room temperature. The solvent was removed in vacuo. The resulting cloudy residue was stirred with 10 ml of toluene for 1 h, then LiCl was filtered off through celite. The obtained orange oil was recrystallized from toluene to afford 0.783 g (69%) of **2** as colorless crystals. Analytic and spectroscopic data of **2**: mp: 168°C, [α] -18.29 (2.8 M in hexane); ^31^P-NMR (CDCl_3_): δ 171.3; ^1^H-NMR (CDCl_3_): δ 0.89-0.68 (6H,m), 0.96 (3H,s), 1.00 (1H,t), 1.05 (3H,s), 1.17 (1H, t), 1.28 (3H, s), 1.43 (3H, s), 1.51-1.49 (1H, m), 1.85-1.66 (2H, m), 7.19-7.15 (1H, d), 7.37-7.26 (2H,m), 7.47 (1H,d). ^13^C-NMR (CDCl_3_): δ 14.54, 15.68, 23.08, 24.35, 24.53, 25.79, 26.80, 27.34, 32.01, 33.07, 33.40, 37.39, 38.75, 42.88, 49.20, 51.34, 124.34, 126.22, 132.26, 134.28, 134.78, 144.22.

**X-ray crystal data of 2**: C_32_H_40_BrO_2_P; M_r_ 567.54, space group: P 2_1_2_1_2_1_ orthorhombic, a = 10.593(1) Å, b = 16.085(1) Å, c = 18.885(1) Å; V = 3217.8(4) Å^3^; Z = 4; T = 100(2) K; µ = 1.351 mm^-1^; reflections total: 14519, unique: 7042, observed: 4848 (*I*>2σ(*I*)); R1 = 0.0518, wR2 = 0.1596; GOF = 1.086.


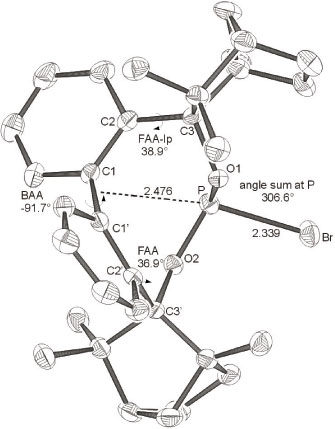


**Figure 2**. X-ray structures of **BIFOP-Br** (**2**). Distances are given in Å. (BAA = biaryl angle between C2-C1-C1’-C2’; FAA-lp = fenchyl-aryl dihedral angle between C1-C2-C3-O1; FAA = fenchyl-aryl dihedral angle between C1’-C2’-C3’-O2). The probability of ellipsoids is 30% (CCDC 270532).

**BIFOP-H (3)**: To 1.0 g of BIFOP-Cl (**1,** 1.9 mmol) in 25 ml hexanes, 5 ml of a 1 M solution of lithium aluminum hydride in THF (5 mmol) were added slowly at room temperature. The mixture then was stirred for 12 h at 69°C. The solvent was removed in vacuo, the residue was taken up in 20 ml of toluene and was stirred for 1h at room temperature. Filtration through celite removed LiCl and other salts. The resulting solution was concentrated in vacuo until precipitation. Recrystallization from toluene yielded 0.739 g (79 %) of **3** as colorless crystals. Analytic and spectroscopic data of **3**: mp: 179.1°C; [α] -38.57 (1.4 M in hexane); IR: ν(P-H) = 2274(s) cm^-1^, ^31^P-NMR (CDCl_3_): δ 139.67; ^1^J(P-H) = 214.45 Hz; ^1^H-NMR (CDCl_3_): δ 0.076 (1H,s), 0.300 (3H,s), 0.500 (3H,s), 0.666 (3H,s), 0.742 (3H,s), 1.190-1.590 (10H,m), 1.619 (3H,s), 1.678 (3H,s), 1.826 (2H,t), 2.393 (2H,t), 6.862 (1H,d), 6.917 (1H,d, ^1^J(P-H) = 214.4 Hz), 7.047 (2H,t), 7.146 (1H,d), 7.183 (2H,t), 7.565 (1H,t). ^13^C-NMR (CDCl_3_): δ 20.26, 22.04, 23.80, 24.00, 28.85, 29.22, 36.39, 36.48, 36.58, 36.64, 44.99, 46.17, 48.58, 49.53, 53.87, 55.88, 92.99, 95.31, 123.98, 124.21, 124.45, 124.84, 128.94, 129.51, 133.01, 134.55, 141.25, 141.87, 145.24, 146.76.

**X-ray crystal data of** **3**: C_32_H_41_O_2_P; M_r_ 488.64, space group: P 2_1_ monoclinic, a = 8.0016(4) Å, b = 13.9613(15) Å, c = 12.2640(11) Å; β=107.4°; V = 1307.1(19) Å^3^; Z = 2; T = 100(2) K; µ = 0.133 mm^-1^; reflections total: 6214, unique: 4642, observed: 3508 (*I*>2σ(*I*)); R1 = 0.0459, wR2 = 0.0833; GOF = 0.959.


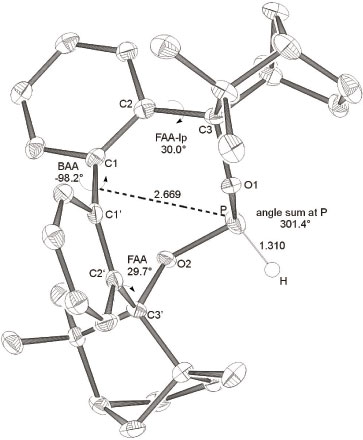


**Figure 3**. X-ray structures of **BIFOP-H** (**3**). Distances are given in Å. (BAA = biaryl angle between C2-C1-C1’-C2’; FAA-lp = fenchyl-aryl dihedral angle between C1-C2-C3-O1; FAA = fenchyl-aryl dihedral angle between C1’-C2’-C3’-O2). The probability of ellipsoids is 40% (CCDC 270533).

**BIFOP-Et (4):** To a solution of 1.0 g of BIFOP-Cl (**1**, 1.9 mmol) in 40 ml hexanes 8 ml of a ethyllithium solution in benzene/cyclohexane (90/10, 0.5 M, 4 mmol) were added. The mixture was refluxed (ca. 65°C) for 24 h. The solvent was removed in vacuo, the residue was taken up in 30 ml of toluene and this mixture was stirred for 1 h. Filtration through celite removed LiCl. Purification by flash chromatography (hexane) and recrystallization from pentane furnished **4** as colorless solid (0.598 g, 63 %). Analytic and spectroscopic data of **4**: mp: 135.9°C; [α] -43.25 (2.6 M in hexane); ^31^P-NMR (CDCl_3_): δ 167.47; ^1^H-NMR (CDCl_3_): δ 0.65 (6H,s), 0.71 (6H,s), 1.11-0.97 (10H,m), 1.37-1.24 (4H,m), 1.71-1.56 (4H,m), 2.22‑2.15 (2H,m), 2.39 (1H,d), 2.43 (1H,d), 2.85 (3H,d), 6.92 (2H, d), 7.11 (2H,t), 7.22 (2H,t), 7.59 (2H,d); ^13^C-NMR (CDCl_3_): δ 17.57, 21.22, 23.71, 30.05, 34.05, 42.53, 42.92, 45.29, 46.49, 49.20, 54.72, 61.36, 69.16, 71.21, 86.14, 91.36, 124.36, 124.77, 129.94, 131.11, 141.22, 144.08.


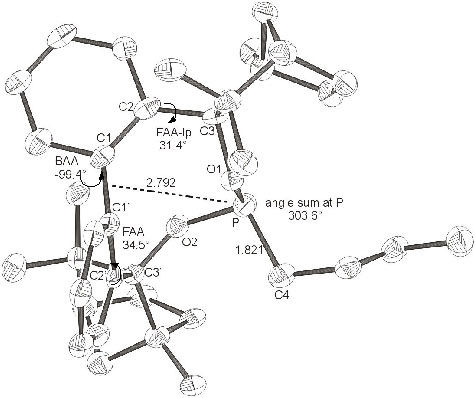


**Figure 4**. X-ray structures of **BIFOP-*n*Bu** (**5**). Distances are given in Å. (BAA = biaryl angle between C2-C1-C1’-C2’; FAA-lp = fenchyl-aryl dihedral angle between C1-C2-C3-O1; FAA = fenchyl-aryl dihedral angle between C1’-C2’-C3’-O2). The probability of ellipsoids is 40% (CCDC 270534).

**BIFOP-*n*Bu (5):** To a solution of 1.0 g BIFOP-Cl (**1**, 1.9 mmol) in 30 ml hexane 1.9 ml n-butyllithium in hexanes (1.6 M, 3 mmol) were added slowly. The mixture was refluxed for 24 h, then the solvent was removed in vacuo. The residue was taken up in 20 ml of toluene, the suspension was stirred for 1 h and then LiCl was filtered off through celite. The product (0.763 g, 74 %) was obtained after crystallisation in diethylether forming colorless needles. Analytic and spectroscopic data of **5**: mp: 130.8°C; [α] -74.31 (4.5 M in hexane); ^31^P-NMR (CDCl_3_): δ 166.9; ^1^H-NMR (CDCl_3_): δ 0.85-0.74 (6H,m), 1.29‑0.98 (12H,m), 1.47‑1.40 (4H,m), 1.67‑1.54 (8H,m), 1.84–1.78 (4H,m), 2.03 (3H,d), 4.86 (2H,d), 7.09‑7.04 (2H,m), 7.15‑7.11 (2H,m), 7.24 (1H, d), 7.28 (1H,d), 7.44 (1H,d), 7.47 (1H,d); ^13^C-NMR (CDCl_3_): δ 13.61, 20.61, 21.37, 22.05, 22.37, 22.59, 23.43, 23.65, 26.88, 27.29, 27.64, 28,39, 29.63, 33.27, 38.72, 42.68, 45.10, 45.42, 49.43, 51.25, 52.25, 63.26, 106.10, 123.940, 124.16, 124.88, 125.04, 132.87, 133.01, 133.16, 133.68, 136.43, 145.83, 160.62.

**X-ray crystal data of 5**: C_36_H_49_O_2_P; M_r_ 544.75; space group: P 2_1_ monoclinic; a = 10.989(1) Å, b = 11.462(1) Å, c = 11.786(1) Å; β=92.25 °; V = 1483(2) Å^3^; Z = 2; T = 100(2) K; µ = 0.124 mm^-1^; reflections total: 6284, unique: 5074, observed: 4349 (*I*>2σ(*I*)); R1 = 0.0483, wR2 = 0.1137; GOF = 1.043.

**BIFOP-O-Ph (6):** To a solution of 1.0 g BIFOP-Cl (**1**, 1.9 mmol) in 25 ml abs. hexane, 2.7 g lithium phenolate (21 mmol, prepared from phenol and nBuLi 1.6 M in hexane (13.1 ml, 21 mmol)) was added slowly at room temperature and stirred in reflux for 24 h. The solvents was removed in vacuo, 50 ml toluene was added and stirred for 1 h at rt. The LiCl was filtered through celite. Purification of the residue by flash chromatography (hexane) followed by crystallization from pentane furnished the title compound as a colorless crystals (0.494 g, 47 %). Analytic and spectroscopic data of **6**: mp: 124.8°C; [α] -20.05 (1.9 M in hexane); ^31^P-NMR (CDCl_3_): δ 129.96; ^1^H-NMR (CDCl_3_): δ 0.50 (1H,s), 0.69-0.64 (1H,m), 0.83 (3H,s), 0.94-0.92 (4H,m), 1.02-0.99 (4H,m), 1.08 (3H,s), 1.51-1.20 (8H,m), 1.80-1.62 (3H,m), 2.00-1.90 (1H,m), 2.20-2.14 (1H, m), 2.55-2.28 (2H,m), 2.79(1H,d), 7.08-7.00(3H,m), 7.32-7.15 (6H,m), 7.56 (1H,d), 7.67 (1H,d), 8.42 (1H,d); ^13^C-NMR (CDCl_3_): δ 15.30, 16.07, 18.22, 22.36, 23.17, 23.97, 24.15, 25.40, 26.44, 26.46, 33.03, 36.01, 38.37, 42.35, 42.49, 44.13, 48.81, 49.54, 52.12, 53.71, 123.84, 123.96, 124.38, 125.28, 125.84, 126.54, 129.37, 131.87, 133.03, 133.44, 133.69, 134.39, 135.16, 137.59, 143.17, 143.89.

**X-ray crystal data of** **6**: C_38_H_45_O_3_P; M_r_ 580.71; space group: P 2_1_ monoclinic; a = 11.038(1) Å, b = 9.933(1) Å, c = 14.412(1) Å; β=100.73°; V = 1552(2) Å^3^; Z = 2; T = 100(2) K; µ = 0.125 mm^-1^; reflections total: 8079, unique: 6071, observed: 5279 (*I*>2σ(*I*)); R1 = 0.0364, wR2 = 0.0741; GOF = 1.022.


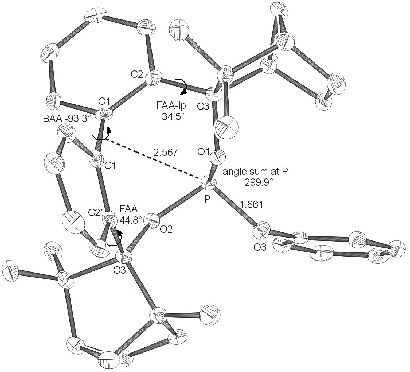


**Figure 6**. X-ray structures of phosphite **BIFOP-OPh** (**6**). Distances are given in Å. (BAA = biaryl angle between C2-C1-C1’-C2’; FAA-lp = fenchyl-aryl dihedral angle between C1-C2-C3-O1; FAA = fenchyl-aryl dihedral angle between C1’-C2’-C3’-O2). The probability of ellipsoids is 40% (CCDC 270535).

***M*-BIFOP-NEt_2_ (7):** To a solution of lithium diethylamide, prepared from diethylamine (2.1 ml, 20 mmol) in 10 ml hexane and *n*BuLi 1.6 M in hexane (12.5 ml, 20 mmol), 3h at rt, BIFOP-Cl (**1**, 1.00 g, 1.9 mmol) in 35 ml abs. hexane was added slowly and then refluxed for 24 h. The solvents was removed in vacuo, 50 ml toluene was added and stirred for 1 h. The LiCl was filtered through celite. The solvent was removed in vacuo, and purification of the residue by flash chromatography (hexane) furnished the title compound as a colorless crystals (0.501 g, 47 %). Analytic and spectroscopic data of **7**: mp: 219.9°C; [α] -119.46 (2.2 M in hexane); ^31^P-NMR (CDCl_3_): δ 126.42; ^1^H-NMR (CDCl_3_): δ 0.705 (3H,s); 0.757 (3H,t); 1.158 (11H,m); 1.413 (3H,m); 1.757 (3H,m); 2.243 (1H, t); 2.473 (1H, d); 2.908 (1H, s); 6.963 (1H, d); 7.148 (1H, t); 7.266 (1H, t); 7.637 (1H, d); ^13^C-NMR (CDCl_3_): δ 17.62, 17.90, 21.27, 23.76, 30.10, 30.59, 34.09, 34.30, 42.23, 42.56, 46.52, 49.23, 49.46, 54.74, 86.16, 86.88, 123.83, 124.40, 129.95, 141.24, 144.09, 145.0.

**X-ray crystal data of** **7**: C_36_H_50_NO_2_P; M_r_ 559.74; space group: P 2_1_ monoclinic; a = 10.993(1) Å, b = 12.774(1) Å, c = 11.073(1) Å; β=98.02°; V = 1539.7(2) Å^3^; Z = 2; T = 100(2) K; µ = 0.122 mm^-1^; reflections total: 9196, unique: 6092, observed: 4692 (*I*>2σ(*I*)); R1 = 0.0432, wR2 = 0.0696; GOF = 0.961.


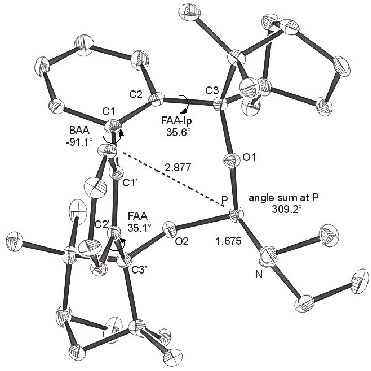


**Figure 7**. X-ray structures of phosphoramidite **BIFOP-NEt2** (**7**). Distances are given in Å. (BAA = biaryl angle between C2-C1-C1’-C2’; FAA-lp = fenchyl-aryl dihedral angle between C1-C2-C3-O1; FAA = fenchyl-aryl dihedral angle between C1’-C2’-C3’-O2). The probability of ellipsoids is 40% (CCDC 270536).

**BIFOP(O)-H (8):** To solid BIFOP-Cl (**1**, 5,33 g, 10,2 mmol) was added 50 ml H_2_O and 1 g KOH. The mixture was refluxed for 5 days. The product was filtered and dried in vacuo. After recrystallization from diethylether the product was obtained as colorless crystals (5.06 g, 98 %). Analytic and spectroscopic data of **8**: mp: 199.6°C; [α] -46.93 (1.4 M in hexane); ^31^P-NMR (CDCl_3_): δ 2.54; ^1^J(P-H) = 700.2 Hz. ^1^H-NMR (CDCl_3_): δ 0.257 (3H,s); 0.48 (3H,s), 0.67 (3H,s), 0.73 (3H,s), 1.53 (3H,s), 1.34-1.48 (3H,m), 1.61-1.75 (8H,m). 5.48 (1H,d ^1^J(H-P)=700.2 Hz), 6.85 (1H,d), 7.09 (1H,t), 7.19‑7.28 (4H,m), 7.48 (1H,d), 7.64 (1H,d); ^13^C-NMR (CDCl_3_): δ 18.91, 20.25, 21.12, 21.98, 23.43, 23.63, 28.55, 28.96, 35.29, 35.37, 44.33, 45.12, 49.01, 50.01, 48.62, 50.14,  48.74, 50.28, 96.326, 96.43, 124.66, 125.11, 125.67, 125.94, 128.27, 128.70, 133.83, 135.81, 138.77, 138.88, 143.18, 145.04;

**X-ray crystal data of** **8**: C_32_H_41_O_3_P; M_r_ 504.62; space group: P 2_1_2_1_2_1_ orthorohombic; a = 8.146(1) Å, b = 14.091(1) Å, c = 23.058(1) Å; V = 2646.7(4) Å^3^; Z = 4; T = 100(2) K; µ = 0.136 mm^-1^; reflections total: 15432, unique: 5722, observed: 3860 (*I*>2σ(*I*)); R1 = 0.0702, wR2 = 0.1644; GOF = 1.068.


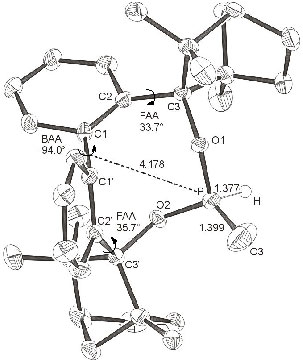


**Figure 5**. X-ray structures of **BIFOP(O)-H** (**8**). Distances are given in Å. (BAA = biaryl angle between C2-C1-C1’-C2’; FAA-lp = fenchyl-aryl dihedral angle between C1-C2-C3-O1; FAA = fenchyl-aryl dihedral angle between C1’-C2’-C3’-O2). The probability of ellipsoids is 40% (CCDC 270537).

**BIFOP(O)-Cl (9):** To solution of biphenyl-2,2'-bisfenchol (BIFOL,^[Error: Reference source not found]^ 0.917 g, 2 mmol) in hexane (25 ml) nBuLi (2.45 ml, 4 mmol) was added slowly at 0°C and stirred for 1 h. POCl_3_ (0.18 ml, 2 mmol) was added and the mixture was stirred for 18 h at rt. The solvents was removed in vacuo, 20 ml toluene was added and stirred for 1 h. LiCl was filtered through celite. The solvent was removed in vacuo and the product (0.701g, 65%) was obtained after crystallisation from acetone. Analytic and spectroscopic data of **9**: mp: 139.2°C; [α] -116.68 (2.8 M in hexane); ^31^P-NMR (CDCl_3_): δ -9.858; ^1^H-NMR (CDCl_3_): δ 0.29 (1H,s), 0.50 (1H,s), 0.71-0.66 (9H,m), 0.86 (1H,m), 1.11-0.97 (6H,m), 1.75-1.33 (12H,m), 2.43-2.39 (2H,m), 2.86 (1H,t), 6.89 (1H,d), 6.92 (1H,d), 7.11 (1H,t), 7.28-7.20 (3H,m), 7.67-7.56 (2H,m); ^13^C-NMR (CDCl_3_): δ 16.63, 16.72, 20.31, 22.81, 27.94, 29.15, 33.14, 41.59, 41.63, 44.58, 45.59, 48.30, 53.82, 55.30, 123.46, 123.89, 127.22, 129.03, 130.21, 133.37, 140.32, 143.18.

**X-ray crystal data of** **9**: C_32_H_40_ClO_3_P * ½(C_3_H_6_O); M_r_ 568.1; space group: P 4_3_2_1_2 tetragonal; a = 12.2700(5) Å, b = 12.2700(5) Å, c = 37.594(3) Å; V = 5659.9(6) Å^3^; Z = 8; T = 100(2) K; µ = 0.274 mm^-1^; reflections total: 18530, unique: 6103, observed: 3847 (*I*>2σ(*I*)); R1 = 0.0629, wR2 = 0.1420; GOF = 0.963.


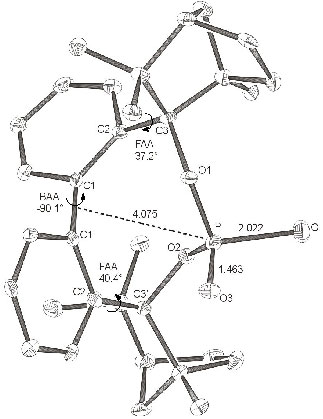


**Figure 8**: X-ray structures of **BIFOP(O)-Cl** (**9**). Distances are given in Å. (BAA = biaryl angle between C2-C1-C1’-C2’; FAA-lp = fenchyl-aryl dihedral angle between C1-C2-C3-O1; FAA = fenchyl-aryl dihedral angle between C1’-C2’-C3’-O2). The probability of ellipsoids is 40% (CCDC 270538).

**Catalytic enantioselective conjugate additions**

A three necked flask was charged with Cu(OTf)_2_ (9 mg, 0.025 mmol), CH_2_Cl_2_, (3 ml) and the BIFOP ligand **1** - **9** (0.05 mmol), respectively. The mixture was stirred for 30 min, then cooled to -40 °C and diethyl zinc (7 ml of a 1 M solution in hexane, 7 mmol) was added. The solution was warmed to -20°C and a solution of 2-cyclohexen-1-one (0.49 ml, 5 mmol) in CH_2_Cl_2_ (3 ml) was added dropwise. The mixture was stirred for 3 h and then quenched by the careful addition of 2 M hydrochloric acid (ca. 5 ml). Diethylether (10 ml) was added at 0°C and the resulting mixture was stirred. The organic phases were dried over MgSO_4_, filtered off and the solvent was removed in vacuo. After flash chromatography (silica, hexan/EtOAc, 80/20) the pure 3-ethyl-cyclohexanone was obtained. The enantiomeric excess was determined by gaschromatography (chiral column: lipodex E, 50 m, T = 81°C, t_R_ = 30 min (R) enantiomer, t_S_ = 32.5 min (S) enantiomer).^[Error: Reference source not found]^ Spectroscopic data of 3-ethyl-cyclohexanon: ^1^H-NMR (CDCl_3_): δ 0.50-0.63 (3H,m); 0.99-1.15 (3H,m); 1.28-1.44 (2H,m); 1.58-1.78 (3H,m); 1.95-2.21 (3H,m); ^13^C-NMR (CDCl_3_): δ 11.15 (C-8); 25.11 (C-7); 29.11 (C-4); 30.71 (C-5); 40.69 (C-3); 41.25 (C-6); 47.59 (C‑2); 211.55 (C-1).

**
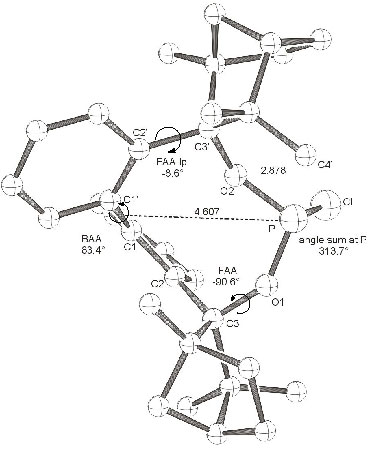
**

**Figure 9**. Computed geometry (PM3) of *plus*-(*P*)-**BIFOP-Cl** with *unnatural plus*-(*P*)-biaryl conformation. Distances are given in Å. (BAA = biaryl angle between C2-C1-C1’-C2’; FAA-lp = fenchyl-aryl dihedral angle between C1-C2-C3-O1; FAA= fenchyl-aryl dihedral angle between C1’- C2’-C3’-O2).
